# Cost-of-Illness Analysis of Type 2 Diabetes Mellitus in Iran

**DOI:** 10.1371/journal.pone.0026864

**Published:** 2011-10-31

**Authors:** Mehdi Javanbakht, Hamid R. Baradaran, Atefeh Mashayekhi, Ali Akbar Haghdoost, Mohammad E. Khamseh, Erfan Kharazmi, Aboozar Sadeghi

**Affiliations:** 1 Health Economics Department, School of Health Care Management, Tehran University of Medical Sciences, Tehran, Iran; 2 Center for Non-Communicable Diseases Control, Ministry of Health Iran, Tehran, Iran; 3 Endocrine Research Center (Firoozgar), Tehran University of Medical Sciences, Tehran, Iran; 4 School of Management and Information Sciences, Shiraz University of Medical Sciences, Shiraz, Iran; 5 Research Center for Modeling in Health, Kerman University of Medical Sciences, Kerman, Iran; Brigham & Women's Hospital - Harvard Medical School, United States of America

## Abstract

**Introduction:**

Diabetes is a worldwide high prevalence chronic progressive disease that poses a significant challenge to healthcare systems. The aim of this study is to provide a detailed economic burden of diagnosed type 2 diabetes mellitus (T2DM) and its complications in Iran in 2009 year.

**Methods:**

This is a prevalence-based cost-of-illness study focusing on quantifying direct health care costs by bottom-up approach. Data on inpatient hospital services, outpatient clinic visits, physician services, drugs, laboratory test, education and non-medical cost were collected from two national registries. The human capital approach was used to calculate indirect costs separately in male and female and also among different age groups.

**Results:**

The total national cost of diagnosed T2DM in 2009 is estimated at 3.78 billion USA dollars (USD) including 2.04±0.28 billion direct (medical and non-medical) costs and indirect costs of 1.73 million. Average direct and indirect cost per capita was 842.6±102 and 864.8 USD respectively. Complications (48.9%) and drugs (23.8%) were main components of direct cost. The largest components of medical expenditures attributed to diabetes's complications are cardiovascular disease (42.3% of total Complications cost), nephropathy (23%) and ophthalmic complications (14%). Indirect costs include temporarily disability (335.7 million), permanent disability (452.4 million) and reduced productivity due to premature mortality (950.3 million).

**Conclusions:**

T2DM is a costly disease in the Iran healthcare system and consume more than 8.69% of total health expenditure. In addition to these quantified costs, T2DM imposes high intangible costs on society in terms of reduced quality of life. Identification of effective new strategies for the control of diabetes and its complications is a public health priority.

## Introduction

Type 2 diabetes mellitus (T2DM) is a chronic disease characterized by hyperglycemia and dyslipidemia due to underlying insulin resistance. The condition commonly progresses to include microvascular [e.g., retinopathy, nephropathy, and neuropathy] and macrovascular [e.g., heart, cerebral, and peripheral vascular disease] complications [1], [2].

The risk of diabetes continues to increase worldwide and its public health burden is unevenly distributed across socioeconomic strata [3], [4]. This burden is not only related to health care costs, but also to indirect costs caused by loss of productivity from disability and premature mortality [5]. The most recent data from the International Diabetes Federation (IDF) indicate that diabetes currently affects 246 million people worldwide and is expected to affect 380 million by 2025 [6]. The Middle East is expected to bear one of the world's greatest increases in the absolute burden of diabetes in the coming decades [7].

According to IDF estimations global health expenditures to prevent and treat diabetes and its complications was at least 376 billion US dollar (USD) in 2010. By 2030, this number will exceed 490 billion USD. There is a large disparity in healthcare spending on diabetes between regions and countries. More than 80% of the global expenditures on diabetes are made in the world's economically richest countries, not in the low- and middle-income countries where 80% of people with diabetes will soon live [6].

However, the ultimate goal of prevention of T2DM is to improve the prognosis and overall quality of life of affected individuals, and with regard to health policy, also to prevent increasing costs of the treatment of T2DM and its complications [8]. Knowledge of the costs of diabetes improves understanding of the importance of addressing health care and prevention issues associated with diabetes. Nonetheless one of the serious handicaps for researchers intending to evaluate alternative interventions for the management of diabetes is scarcity of cost data especially on T2DM [9]. Since there are few studies regarding cost of diabetes in Iran and Middle East countries, particularly about T2DM and its complications, therefore the aim of this study is to give a better economic perspective of cost of T2DM in Iran for health policy making.

## Methods

### Study design

This is a prevalence-based cost of illness (COI) study focusing on direct health care costs. A multipoint data collection procedure based on the patient medical records beside diabetes prevalence rates and other epidemiological data, health care costs, and economic data used in order to obtain the necessary data for the analysis and the construction of Cost of T2DM Model. The perspective for this study was society of Iran. As well as direct costs, the analysis incorporated indirect costs. These costs included loss of productivity due to temporarily and permanent disability and labor loss due to premature mortality [10].

### Study Subjects and Sampling Design

As a part of a national survey of diabetes prevention and control programme, using a multistage cluster randomized sampling method, 4500 subjects were recruited from Tehran and Fars province, capital and south of Iran respectively with a population of around 18.5 million (about 25.5% of the Iranian total population). Each province as strata of study sample has seven clusters for urban and rural regions, which were selected randomly. To produce the target sample size, subjects were selected using the probabilities proportional to their region population. Subjects were eligible for the study if at the first they met WHO criteria (fasting plasma glucose ≥7.0 mmol/l (126 mg/dl) or with a glucose tolerance test, two hours after the oral dose a plasma glucose ≥11.1 mmol/l (200 mg/dl)). Second at least they received routine care of diabetic patients included: regular visits for glycemic control and screening for complications, regular laboratory investigations and medications for one year. Third were age 18 years or older and forth were willing and able to give written informed consent and complete the questionnaire interview that composed of the following sections: **Section A**- demography and background information, **Section B**- diabetic care, **Section C**- outpatient care, **Section D**- inpatient care and **Section E**- informal care and other direct non medical cost and indirect cost. General Characteristics of Subjects showed in table 1.

**Table 1:** General Characteristics of Subjects.

Characteristic category	Subcategory	Number (%)
**Province**	Tehran	3424 (76)
	Fars	1076 (24)
**Region**	Urban	3561 (79)
	Rural	939 (21)
**Sex**	Male	1980 (44)
	Female	2520 (56)
**Age**	20–29	54 (1.2)
	30–39	463 (10.3)
	40–49	1116 (24.8)
	50–59	1611 (35.8)
	60–69	896 (19.9)
	>70	360 (8)
**Insurance**	Social Insured	4212 (94)
	Private insured	83 (1.4)
	Uninsured	205 (4.6)
**Employment status**	Employed	1591 (35.4)
	Student, housewives, voluntary workforce	2480 (55.1)
	unemployed	428 (9.5)
**Education**	University education	1028 (22.8)
	Non entrance university	950 (21.1)
	Illiterate	2522 (56)

### Determining direct medical costs

The approach used to estimate medical costs largely follow the investigating medical records of people with diagnosed T2DM -were registered in several participating centers- by bottom-up approach. All patients attended diabetes clinics every 2 or 3 months for the duration of the study. At each visit they were precisely assessed to determine the occurrence of any clinical events or hospital episodes since the previous visit. Full clinical and cost information on hospital in-patient stays was collated by each clinic based on patient interviews at every clinic visit. Information on non-in-patient health care resources was obtained using a questionnaire (section c) distributed at clinic visits during the survey period. This survey recorded information on all contacts with general practitioners, nurses, podiatrists, opticians, and dieticians, other hospital out-patient clinics and any other pharmaceutical, diagnostic and rehabilitative care received during the survey. The total costs over this period were estimated by multiplying the number of each care by unit costs for each type of care and the resulting total cost was then annualized. To investigate the distribution of costs across each cost component, resource utilization was separated in to six categories: costs of visits to specialists; pharmaceutical and device, laboratory/diagnostic tests, inpatient and educational cost for patients without complication and costs associated with complications. All health care resources utilization categorized for patients with and without complication. The estimated aggregate total annual direct medical costs associated with T2DM and its complications were calculated for the population of Iran on the basis of age and sex-specific prevalence rates of T2DM and its complications (table 2).

**Table 2:** Diabetes-related complications included in the regression analysis, with diagnostic definitions and theirs prevalence in Iran.

Complication	ICD-9	Prevalence	Reference
**Cardiovascular disease**	401–405, 410, 411, 412, 413, 414–414.8	28%	[11]
**Neuropathy**	250.6	41.60%	[12]
**Peripheral vascular disease**	440, 443, 451,452,454, 459.8–459.9,	14.30%	[13]
**Nephropathy**	580–586	25.60%	[14]
**Ophthalmic complications**	362.01–362.07, 366, 365	37.00%	[15]
**Diabetic foot**	736, 707	4%	[16]

### Determining direct nonmedical costs

Non-medical expenditures include costs for services such as transportation for the patient and family members to clinics that estimated from the patient's self-estimate questionnaire and costs for taking care of dependents (informal care that not included as part of direct health care costs). The cost of T2DM related informal care was estimated using Posnett and Jan's method for measuring the opportunity cost of unpaid inputs [17]. The average net hourly wage was applied to informal care provided by carers under 65 year who were economically active and in employment [18].

### Determining costs of lost productivity

In this study the total indirect costs associated with T2DM categorized in three groups. Health-related days absent from work and reduced job performance due to health problems (Temporarily Disability), reduced earnings capacity from permanent disabilities, and lost productivity from premature mortality. we asked patients number of days in year who couldn't be in their job due to seeking diabetes related health care or rested in home to get better (temporarily disability) in E section of questionnaire. Then we used their insurance ID to retrieve their indemnity claims during one year from social security organization (SSO) database. It should be noted that SSO data would lead to an underestimate of the number of disabled people because it provides data on the number of people who receive disability benefits. The number of people who are actually disabled exceeds the number who receives benefits. So we made some adjustments according to respondents' declarations. Also according to each patient's condition and their complication's disability weight -obtained from SSO data on disability by condition- [19] we calculated annual permanently disability costs. Mortality costs are calculated as the lost earnings due to premature mortality. The number of years of life lost, estimated by using Iran's life table (2009) and number of death in different age and sex group attributable to T2DM. The productivity loss for people employed in the workforce is calculated by combining age-sex–specific estimates of workforce participation rates (estimated from the Statistics Center of Iran (SCI)), average earnings (from the Central Bank of the Iran (CBI)), and the size of the population with diagnosed T2DM. The earnings in future years are discounted (3% discount rate, a rate often used in public health studies) and one percent real annual growth rate in earnings is assumed. We assume that the value of productivity generated for the population age 20–69 who is not employed is 75% of the value of employed people of the same age and sex [10], [17]. Also we assumed that productivity loss cost for population age 70 and older is zero. To have an international perspective both direct and indirect costs were converted from Iranian Rials [IRR], into USD at an official currency exchange rate of 9,920 IRR/1 USD 2009 [20].

### Statistical analysis

Healthcare cost data often have several characteristics that must be addressed through the careful selection of appropriate statistical analyses [9]. Within a defined period (e.g.1 year) significant proportions of individuals have no contact with some types of healthcare providers and so incur no costs. However, amongst the individuals who do make use of health services, the cost data are typically right-skewed because a relatively small proportion of patients incur extremely high costs. Even if the estimate is unbiased from the linear regression models, it could be unstable given the skewness and kurtosis of the data distribution, and inefficient due to the heteroskedasticity [21]. Such problems were dealt with by logarithmic or other transformation of the cost data. We used the generalized linear model (GLM) with log-link and gamma variance functions which assume patient characteristics and complications have a multiplicative effect on costs to identify the relationship between complications and costs after controlling for covariates (including; sex, age and location (urban or rural)). Also we used standardized sampling weights for data analyzing.

At the end because a range of sources and assumptions were used, there is inevitably some uncertainty in the estimated costs of T2DM. Therefore sensitivity analysis was conducted on direct and indirect costs to test the robustness of the assumption and to examine the impact of potential outliers in the claim database. The effects of 20% changes in the baseline resource quantities, unit costs, and other key data on direct health care costs and productivity loss were tested. The effects of changes in discount rates on productivity cost were assessed by using rates of 3% and 5%.

## Results

Total numbers of Iranian people with diagnosed T2DM has been estimated approximately 2.43 million in 2009. The total national cost of T2DM in the Iran is estimated at 3.78 billion [USD]. It consists of direct medical and non-medical costs 2.04±0.28 billion and indirect cost of 1.73 billion. Average medical cost per capita was 842.6±102 USD. The largest components of per capita medical expenditures are complications cost 412.8±64.5 USD (48.9%); drugs 200.6±33 (23.8%), inpatient services 80±12.7 (9.5%) and Laboratory tests 76±11 (9%). About 8.69 percent of total health care expenditure in Iran is attributable to diagnosed T2DM (Table 3).

**Table 3:** Per capita and total direct cost attributable to T2DM [USD].

Resource category	Per capita (USD)	% of Total direct cost	Iran(million USD)	% of Iran's Total Health Expenditure
**Physician visit**	50.9±5.6	6	123.7±11.5	0.52
**Drug prescription**	200.6±33	23.8	487.8±44.1	2.07
**Laboratory use**	76±11	9	184±28	0.78
**Education**	4.2±0.8	0.5	10.2±2.7	0.04
**Inpatient**	80±12.7	9.5	194.5±32.3	0.83
**Complications**	412.4±64.5	48.9	1002.7±140	4.25
**Non medical**	18.5±3.2	2.2	45±9.8	0.19
**Total direct cost**	842.6±102	100	2048.8±280.6	8.69

The cost of complications attributable to diagnosed T2DM accounted for a 1.002±0.14 billion USD in Iran's health system cost (more than 4.25% of Iran's total health expenditure). Per capita direct cost in patients with one or more complications was 2 times higher than those without complications. Cardiovascular disease 424.8±87 million USD, nephropathy 229±49 and ophthalmic complications 140±21.5 are main component of complication cost in both sexes with 42.3%, 23% and 14% respectively. Table 4 shows medical cost of each complication in both sexes separately. Estimated per capita cost of managing neuropathy and nephropathy complications in males are higher than females and this difference was statistically significant (P-value<0.05) however total complications per capita cost was not different.

**Table 4:** Health care expenditures attributed to T2DM complications [USD].

	Per capita cost USD [95% CI]		Total cost million USD [95% CI]			
Medical condition	Male	Female	Male	Female	Both sex	% of Total Complication Cost
**Cardiovascular disease**	158.5 [134.1–182.8]	186.1 [148.2–224.8]	162.9 [128–196.3]	261.2 [208.6– 314.3]	424.8 [336.6–510.1]	42.3
**Neuropathy**	13.7 [11.7–15.8]	3.5 [2.9–4.1]	14 [11.6–16.4]	4.9 [3.8–6]	18.9 [15.4–22.4]	1.9
**Peripheral vascular disease**	31.3 [26.4–36.4]	35.6 [29.8–41.5]	32.1 [27.8–36.4]	49.9 [40.2–59.6]	82.1 [68–96]	8.2
**Nephropathy**	134.8 [108 – 160.5]	64.5 [48.2 – 80.3]	138.2 [103–173.3]	90.5 [76.6–104.5]	229 [179.6–274.8]	23
**Ophthalmic complications**	54.7 [42.3 – 66.5]	59.7 [47.1 – 72.1]	56.2 [47.1–65.3]	83.8 [71.4–96.2]	140 [118.5–161.5]	14
**Diabetic foot**	36.8 [32.2 – 41.3]	49.4 [41.2 – 57.1]	37.8 [31.2–44.4]	69.3 [59.6–78]	107.1 [90.8–122.4]	10.7
**total**	429.8 [374 – 484]	398.5 [352 – 446]	441.7 [382.4–502.1]	559.8 [481–637.4]	1002 [863.4–1139.5]	100

The national cost of lost productivity associated with diabetes is estimated at 1.73 billion USD. This includes temporarily disability (335.7 million), permanent disability (452.4 million) and reduced productivity due to premature mortality (950.3 million). Average per capita indirect cost was 864.8 USD that equal 19 percent of Iranian per capita national income. Based on Iran's SSO data we have concluded that average lost number of workdays per employed person per year is 17.7 included health related absenteeism and productivity loss while at work (Temporarily Disability). As well as these patients lost 23.8 work days due to permanent disability annually. Also according to estimated number of deaths in males and females attributable to T2DM in the 20–69 age-group in Iran by IDF , we calculated total number of work days lost due to premature mortality 152 million days. Temporarily and permanent disability and premature mortality account for 19.3%, 26% and 54.7% of total indirect cost respectively. As showed in table 5 the population with the highest productivity loss due to temporarily and permanent disability and premature mortality are in 40–59, 40–59 and 60–69 age groups respectively.

**Table 5:** Indirect cost of T2DM by age and sex group [USD].

			Total cost [million USD]		
Cost category	Subcategory	Average per capita cost[USD]	Male	Female	Percent of total indirect cost (both sexes)
**Temporarily Disability**	**20–39**		24.3	28.8	3.1
	**40–59**		97.1	115	12.2
	**60–69**		32.2	38.2	4
	**Total (both sexes)**	166.9	335.7		19.3
**Permanently Disability**	**20–39**		32.8	38.9	4.1
	**40–59**		130.9	155.1	16.4
	**60–69**		43.4	51.4	5.5
	**Total (both sexes)**	225	452.4		26
**Premature mortality**	**20–29**		19	10.5	1.7
	**30–39**		45.3	45.5	5.2
	**40–49**		73.4	96.2	9.8
	**50–59**		146.3	178.5	18.7
	**60–69**		173.5	162.2	19.3
	**Total (both sexes)**	472.7	950.3		54.7
**Total indirect cost**		864.8	1738.4		100

In the one way sensitivity analysis, 20% change in assumptions concerning to health care utilization and unit costs of health care used in each complication was not most sensitive to assumptions - resulted in only moderate changes in direct health care cost (15–19% change). We have concluded that present value of future productivity loss due to premature mortality is sensitive to the chosen discount rate, each percentage point increase in the discount rate causing the Iran total productivity loss drop by 5.3 percent (about 92 million).

## Discussion

A synthesis of the published literature showing that one of the world's greatest increases in the absolute burden of diabetes in the coming decades will be happen in Middle East [6], [22]. Most of this increase is anticipated to affect the economically productive 45–64 year old age segment in contrast with most developed countries, where the increase in patients with diabetes will occur in those aged ≥65 years [23].

There is a wide variety of COI on diabetes in developed countries. However, these estimates vary substantially based on what has been left out and what has been included as costs. In addition, it seems almost difficult to compare costs between countries as these costs often represent decisions which are made in each country regarding health care financing and delivery [24] but in order to evaluate the magnitude of the economic burden of diabetes, it is useful to compare the cost estimates of T2DM in Iran with other countries. The results of our analysis indicate that T2DM is a costly disease for Iran's health care system. We estimated total direct cost attributable to T2DM approximately 2.04 billion [USD]. Our study showed that the main component of direct cost in patients without complication was drug (46%) which is in agreement with Esteghamati and his colleague's study [25] that estimated medications and device as a main component of direct cost with 33 percent proportion.

We found that average annual direct cost per patient was 842.6 [USD], while respective findings of published studies performed in United State in 2008 -that concluded, diabetes is responsible for 6,649 dollar in excess expenditures per year per person- [10] revealed that annual per capita direct cost in Iran is about one third of USA one's, - based on purchasing power parities (PPP), which is a method of measuring the relative purchasing power of the currencies of different countries over the same types of goods and services, by eliminating the differences in price levels between countries. Also Estimated average per capita costs of diabetes mellitus in Latin America and the Caribbean were USD 703; highest in Cuba (USD 1219) and lowest in Colombia (USD 442) in 2000 [5]. Similarly a recent study assessing the treatment costs of diabetics in Karachi – Pakistan [26] estimated the annual mean treatment costs per DM patient to be 197. Given that in Iran more than half of annual per capita diabetes cost was out-of-pocket expenditure; this represents a significant burden for the individual with T2DM.

We found that annual per capita cost of T2DM patients was 3.1 times higher than average per capita health care expenditure in Iran (842.6 vs. 269 USD in 2009). While Esteghamti and his colleague estimated that annual per capita direct cost of T2DM were 2.9 time higher than those non diabetic participants in their study [25]. Meanwhile in USA people with diabetes have health care expenditures that are 2.3 times higher than expenditures for this same population would be in the absence of diabetes [10]. According to our result 48.9% of total T2DM direct medical cost is attributable to its complications; in accordance with result of other studies that conducted in Iran and USA [25], [10] and estimated this number 53% and 50% respectively. We concluded that per capita direct cost in patients with one or more complications was about 2 times higher than those without complications, while respective finding in Iran and UAE were 1.88 and 9.4 respectively [25], [27]. We found that cardiovascular disease was main component (42.3%) of complication cost. Jaime Caro and his colleagues estimated that management of macrovascular disease is estimated to be the largest cost component, accounting for 52% of the complication costs [28]. Estimated per capita cost of managing neurological and nephropathy complications in males was higher than females, but total per capita cost in both sexes is not significantly different (table 4). This finding should be interpreted discreetly, since we estimated spending on each complication for male and female in average and difference in expenditure can be due to other reasons such as high prevalence and severity of neuropathy and nephropathy complications in male compared to female.

Costs of Diagnosed T2DM, accounts for about 8.96 percent of Iran's total health expenditure in 2009 (without taking into account other type of diabetes and undiagnosed patients) while a study conducted in Iran in 2005 [25] showed that this proportion was 5.5%. Since health care utilization patterns and economic burden associated with diabetes mellitus is different between diagnosed and undiagnosed patients therefore multiplying per capita cost by total number of diabetic patients (diagnosed and undiagnosed) is not very accurate and suggested. Timothy and his colleague found that annual per capita direct cost in patient with diagnosed diabetes was 5.5 time higher than undiagnosed patient (9677 and 1745 USD) [29], [30]. Given that, about 30 to 50 percent of total diabetic patients in Iran are undiagnosed [31], [23], if people with undiagnosed diabetes have diabetes-related medical costs that are 10, 20, or 30% as high as people with diagnosed diabetes, then the national cost of T2DM could be $136, $273 or $409 million higher, respectively, than our estimates suggest.

Total estimated indirect cost of diabetes in Iran was 1.73 billion. Lost productive time cost that was expressed as an average across all individuals who met criteria for T2DM was 864.8 USD per years. We found that indirect costs accounted for 44.5% of diabetes-related total costs in Iran, whereas in recent studies in USA and Colombia this proportion was 33% and 65.8% respectively [10], [32].

Our findings are robust to all of our base-case assumptions in one-way sensitivity analyses. Some may argue that one way sensitivity analysis could substantially underestimate the uncertainty in cost of illness estimates [18]. To address this concern, the effect of our simultaneous change of all unit costs by ±20% resulted in moderate changes in our estimates of direct health care cost.

The findings in this study may be subject to several limitations such as: absence of a single data source for all estimates, small sample size in some data sources, correlation of both diabetes and its co morbidities with other factors such as age and obesity. Also we did not take into account expenditures for prevention programs targeted to people with diabetes (e.g., disease management programs), research activities (e.g., to develop new drugs), and administration costs (e.g., to administer the national programs, to process insurance claims) in cost estimates due to data limitations. This study did not include undiagnosed diabetes and intangible cost (reduced quality of life, and the pain and suffering of people with diabetes, their families, and their friends) therefore, the health care costs attributable to T2DM have been underestimated. The lost productivity estimates are for those individuals with diagnosed diabetes and exclude lost productivity associated with diabetes of family members. For example, the productivity loss associated with adults who take time off from work to care for an elderly parent is not included in the cost estimates. Moreover, the extrapolation of results from Tehran and Fars to Iran should be interpreted with caution. Tehran and Shiraz seem to be more urbanized than other provinces of country and accessing to health care facilities in these provinces seems to be more than some other province and this may cause overestimate the diabetes cost. In addition we used human capital approach to estimate lost productive time cost but, the hman capital approach may overestimate the costs, while the frictional cost approach give more realistic estimates. The lack of information needed to apply the frictional cost approach cause us to apply first approach.

Many socioeconomic factors and healthcare system related factors influence the outcome of diabetes and consequently its costs. In this context, diabetes care in developing countries is accompanied by certain societal issues patient behaviors, attitudes, and beliefs, alongside issues such as finances and education, hinder effective diabetes control [33]. As diabetes initially presents with few symptoms and is not life-threatening, many people, particularly in developing countries, often do not seek medical attention until other incapacitating symptoms or complications develop [34]. Delay in diagnosis can directly increase complications and then lead to higher direct and indirect costs [25]. Awareness of economic burden related to each complication of T2DM and what drives cost can be very useful in health-system reform to minimizing the long-term economic burden of this growing epidemic.

Finally while diabetes mellitus is increasingly prevalent in the Middle East, the population remains largely unaware of the devastating effect of the disease. It is important both to educate health professionals and the people in the region and to effectively manage the disease in order to ultimately help control the epidemic.

The present findings demonstrate that T2DM and its complications impose significant burden on the individual and health care system in Iran. Much of this cost is preventable through improved diet and exercise and prevention initiatives to reduce the prevalence of diabetes and its comorbidities. Improved understanding of the economic cost of diabetes and the major determinants of costs helps to inform and motivate decisions that can reduce the national burden of this disease.
